# Extramedullary plasmacytoma of the thyroid: a case report and literature review

**DOI:** 10.3389/fonc.2026.1752447

**Published:** 2026-03-13

**Authors:** Ruichen Sun, Dongqing Pu, Rongsi Wan, Xiangxiang Yu, Hanhan Chen, Minmin Yu

**Affiliations:** 1 First Clinical Medical College, Shandong University of Traditional Chinese Medicine, Jinan, China; 2 Department of Thyroid and Breast Diagnosis and Treatment Center, Affiliated Hospital of Shandong University of Traditional Chinese Medicine, Jinan, China; 3 Department of Pathology, Affiliated Hospital of Shandong University of Traditional Chinese Medicine, Jinan, China

**Keywords:** case report, Hashimoto’s thyroiditis, literature review, solitary extramedullary plasmacytoma, thyroid pathology

## Abstract

**Background:**

Thyroid extramedullary plasmacytoma (EMP) is an exceedingly rare malignancy, often coexisting with Hashimoto’s thyroiditis (HT). Due to its nonspecific clinical presentation, it is frequently misdiagnosed. Here, we presented a complete diagnostic workup of a primary thyroid EMP patient, including imaging and comprehensive pathology. Furthermore, we have provided an in-depth literature review of this rare entity.

**Case presentation:**

This is a case of a 70-year-old woman who presented with a neck mass. A fine-needle aspiration before the surgery suggested HT, and intraoperative frozen section rapid pathology suspected medullary thyroid carcinoma. What surprised us was that postoperative immunohistochemistry confirmed the diagnosis of EMP. The patient had favorable outcomes at 6 months after completing the treatment course, which included surgical resection.

**Conclusion:**

Thyroid EMP is highly associated with HT, and many researchers hypothesize that its development may be linked to chronic antigenic stimulation within the inflammatory microenvironment of HT. Cytopathological diagnosis of thyroid EMP can mimic medullary thyroid carcinoma or lymphoma. Therefore, immunohistochemical analysis is mandatory for accurate differentiation. Generally, the prognosis of thyroid EMP is favorable, with recurrence and metastasis being relatively uncommon.

## Highlights

- Thyroid EMP is highly associated with HT, and its development may be driven by chronic antigenic stimulation within the HT-related inflammatory microenvironment.
- The microscopic examination revealed EMP cells with a plasmacytoid morphology, along with clusters of Hurthle cells and stromal amyloid deposits, resulting in a phenotype strikingly similar to that of medullary thyroid carcinoma.
- When diagnosing thyroid EMP, liquid-based cytology is reported to be more accurate than conventional smears. It may offer advantages in diagnostic clarity by providing a cleaner background and better-preserved cell morphology.

## 1 Introduction

Extramedullary plasmacytoma (EMP) is a malignant plasma cell tumor that originates outside the bone marrow (1). It is a rare clinical entity, accounting for only about 3% to 5% of all plasma cell neoplasms (2). EMP is derived from B lymphocytes and is characterized by the monoclonal proliferation of plasma cells, which can arise in any extramedullary tissue throughout the body. Plasmacytoma can present as either an EMP, which arises in soft tissues, or as a solitary bone plasmacytoma (SBP) (3). EMP occurs predominantly in the head and neck region (e.g., nasal cavity, paranasal sinuses, and nasopharynx). However, it has also been rarely reported in other sites such as the soft tissue, gastrointestinal tract, skin, and lymph nodes (4, 5). Among them, a solitary lesion that does not involve the bone marrow is called a solitary extramedullary plasmacytoma (SEP). Primary involvement of the thyroid gland is exceptionally uncommon, representing only about 1% of all SEP cases. Compared to SBP, EMP typically presents as a more localized disease. It is highly responsive to definitive local treatments, which are associated with higher cure rates (6).

The diagnosis of solitary plasmacytoma at extrathyroidal sites using fine-needle aspiration cytology (FNAC) has been documented previously (7). However, to the best of our knowledge, the preoperative FNAC diagnosis of solitary thyroid plasmacytoma remains rare and poses a unique diagnostic challenge. This case underscores the diagnostic difficulty of FNAC in solitary thyroid plasmacytoma, particularly due to the presence of atypical Hurthle cells and associated amyloid within the tumor. These features can closely mimic the cytomorphology and stromal amyloid deposition seen in medullary thyroid carcinoma, leading to potential misinterpretation (8).

It is noteworthy that approximately 60% to 80% of thyroid EMP cases arise against a background of Hashimoto’s thyroiditis (HT), an association too frequent to be considered coincidental. Although the precise pathogenic link between the two conditions remains incompletely elucidated, it is hypothesized that chronic antigenic stimulation within the inflammatory microenvironment of HT may promote aberrant B−cell activation and proliferation, ultimately leading to clonal plasma−cell expansion and the development of thyroid EMP (9). This potential mechanism underscores the importance of clarifying their relationship to inform accurate pathological diagnosis and clinical management.

This study presents the case of a 70-year-old woman with primary thyroid EMP occurring in the setting of HT, detailing the complete diagnostic and therapeutic pathway. The diagnostic process encountered significant challenges: FNAC initially indicated HT, and intraoperative frozen section analysis suggested medullary carcinoma. The definitive diagnosis of EMP was established only through comprehensive postoperative histopathological examination supplemented by immunohistochemistry. A review was conducted of all relevant literature published over the past 34 years to summarize the demographic, clinical, and pathological characteristics of this disease entity. Based on this review, pathogenic hypotheses and differential diagnoses regarding the association between thyroid EMP and HT were further explored.

## 2 Case presentation

### 2.1 Clinical presentation

A 70-year-old woman presented with a two-year history of an anterior neck mass that had rapidly enlarged over the previous three months and was associated with occasional dysphagia. She denied dyspnea, chest tightness, or palpitations. Her past medical history included HT and hypothyroidism, diagnosed two years earlier, for which she was receiving levothyroxine sodium 25 μg daily. She reported no other chronic illnesses and denied any drug or food allergies. On physical examination, the neck appeared thickened with grade III bilateral thyroid enlargement. A firm, well-defined, non-tender mass measuring approximately 10 × 5 cm was palpated in the left lobe, and another mass measuring 15 × 10 cm was detected in the right lobe. Both masses moved with swallowing. No cervical lymphadenopathy was observed.

### 2.2 Preoperative examinations

**Figure 1:**
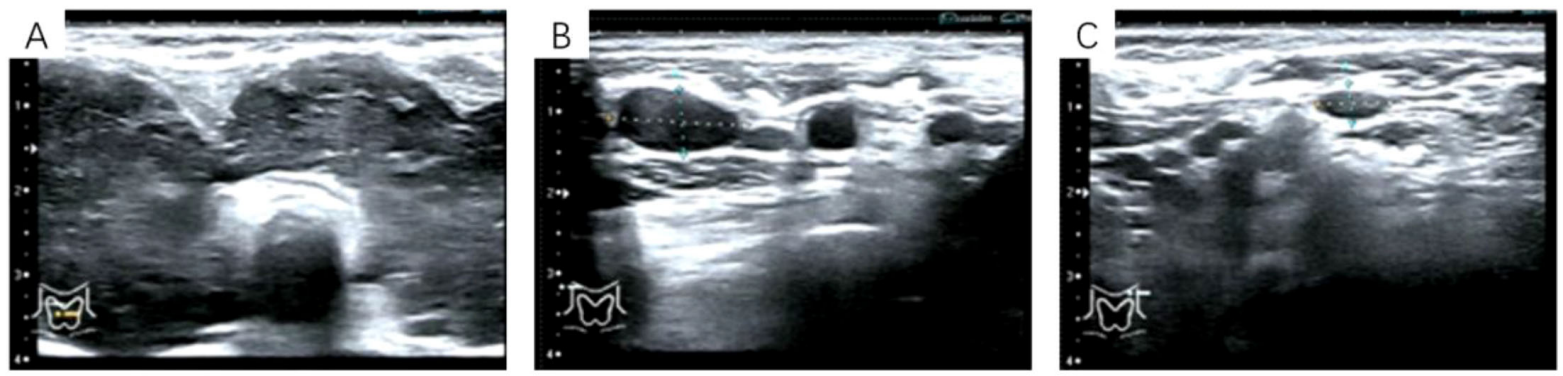
**(A)** Color Doppler ultrasonography of the thyroid. **(B)** Ultrasonography of the right level III cervical lymph node. Dashed lines indicate the dimensions: 9.8 mm (longitudinal) × 4.7 mm (transverse). **(C)** Ultrasonography of the left level III cervical lymph node. Dashed lines indicate the dimensions: 10.3 mm (longitudinal) × 7.5 mm (transverse).

**Figure 2:**
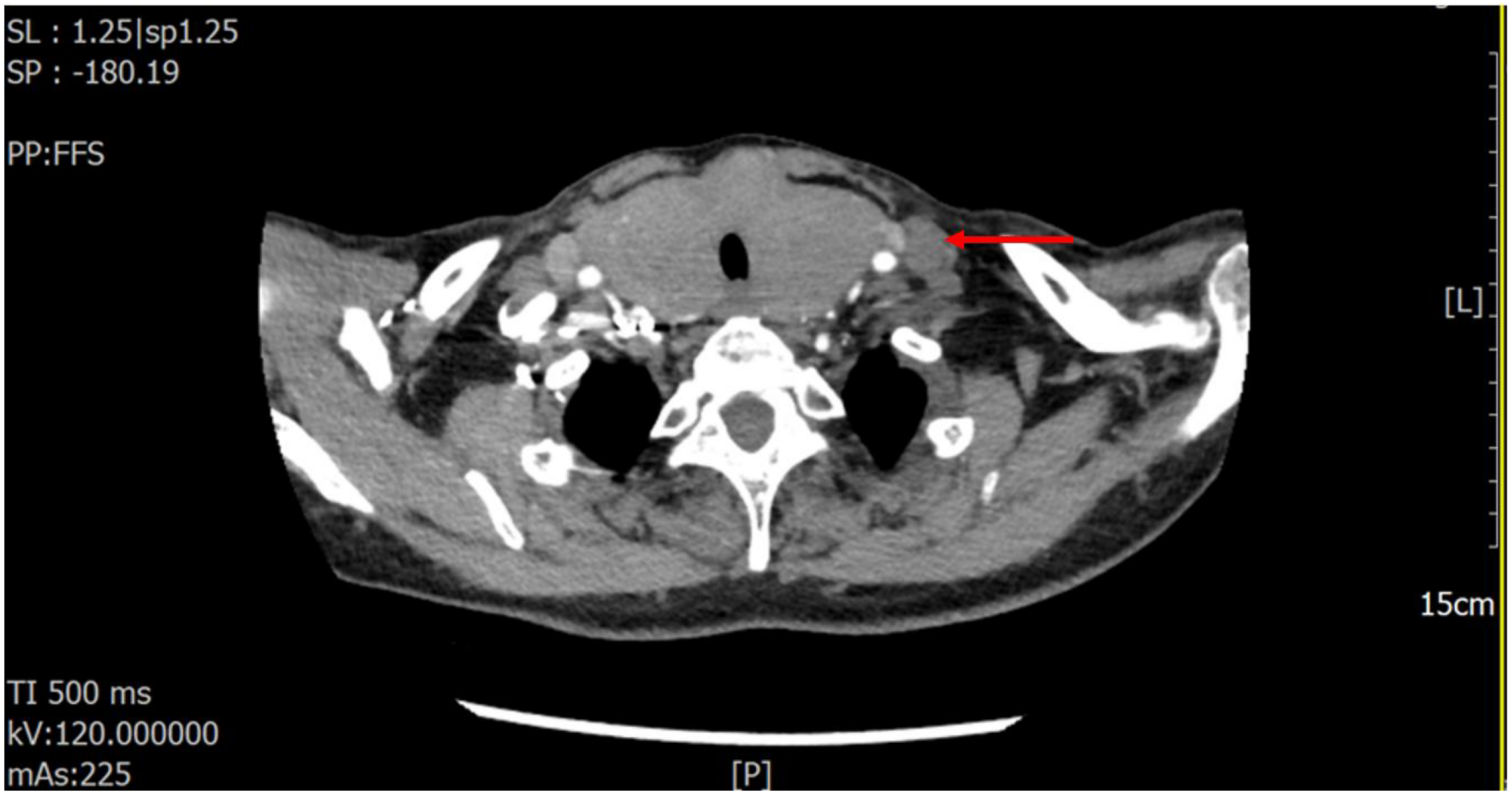
Contrast-enhanced CT of the neck revealing cervical lymphadenopathy (as indicated by the red arrow) and tracheal compression deformity.

Preoperative ultrasonography, contrast-enhanced CT, laboratory tests, and FNAC results were all highly consistent with HT. Thyroid ultrasonography revealed diffuse enlargement and destruction of both lobes, accompanied by bilateral cervical lymphadenopathy. The ultrasound measurements of the thyroid were: right lobe, 34.6 mm; isthmus, 12.7 mm; and left lobe, 31.4 mm. Although the gland maintained a regular shape, it demonstrated a hypoechoic and heterogeneous echotexture. Both lobes extended below the clavicle, resulting in poor visualization of the lower poles and margins (**Figure 1A**). No abnormal intraparenchymal blood flow was detected. Multiple enlarged cervical lymph nodes were observed bilaterally. The largest measured 8.1 × 16.7 mm in level II on the right, 4.7 × 9.8 mm in level III on the right (**Figure 1B**), 4.7 × 8.7 mm in level II on the left, and 7.5 × 10.3 mm in level III on the left (**Figure 1C**). These nodes exhibited indistinct corticomedullary differentiation. Contrast-enhanced CT of the neck (**Figure 2**) demonstrated diffuse thyroid enlargement with reduced density. No significantly enhancing nodules or calcifications were identified. The trachea was compressed and narrowed. Combined with the clinical history, these findings suggested an inflammatory process; however, the possibility of a space-occupying lesion could not be excluded and required further evaluation. Multiple enlarged lymph nodes were also present in the bilateral cervical regions and mediastinum.

**Figure 3:**
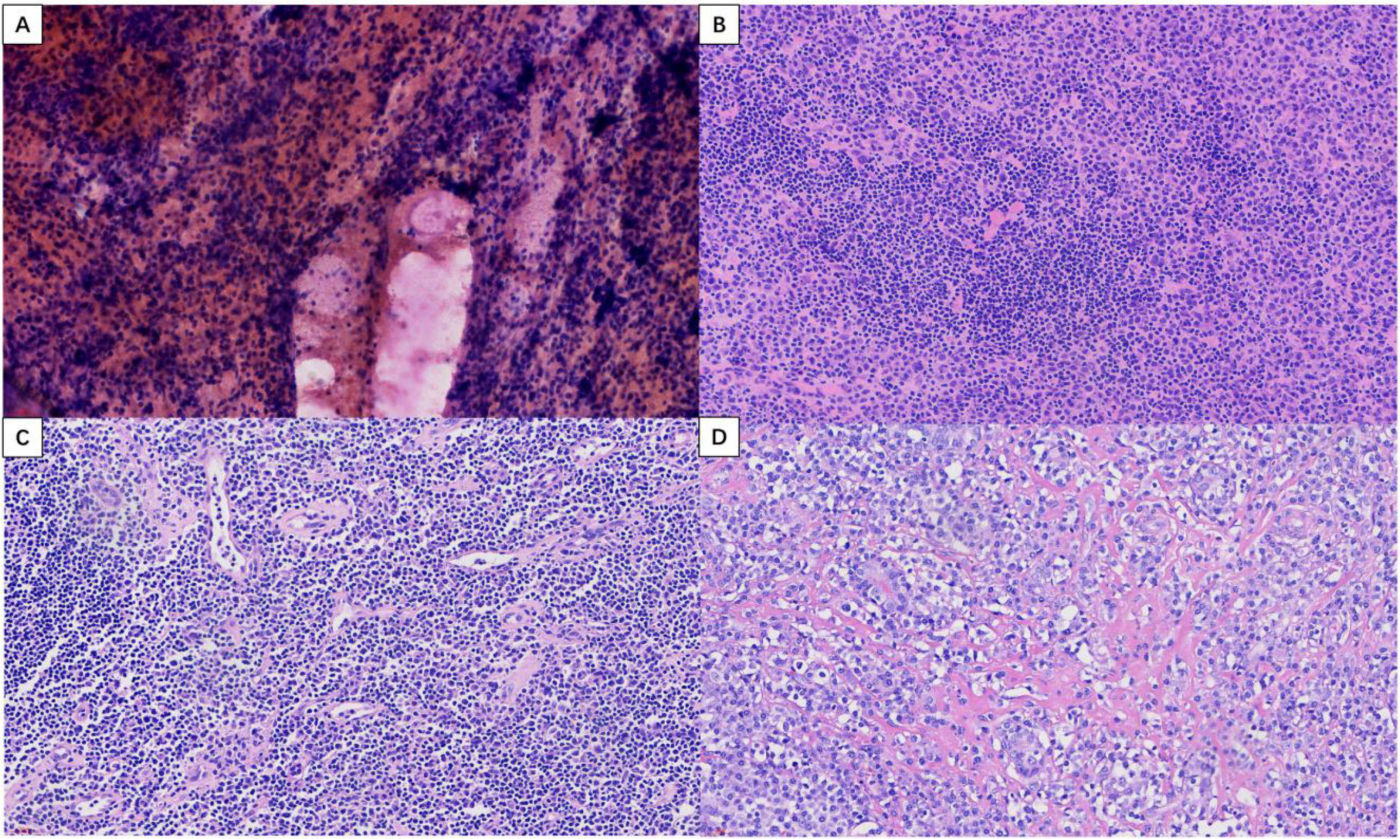
Thyroid FNAC (**(A)**, HE×400), frozen section pathology (**(B)**, HE×400); Lymph node involvement (**(C)**, HE×400), conventional pathology (**(D)**, HE×400).

Laboratory tests showed mildly elevated parathyroid hormone (PTH) at 85.06 pg/mL (reference range: 15–85 pg/mL) and elevated anti-thyroglobulin antibody (TgAb) at 234 IU/mL (reference range: 0–115 IU/mL). Thyroid function tests—including TSH, FT4, FT3, total T4, total T3, and anti-thyroid peroxidase antibody (TPOAb)—were within normal limits, as were complete blood count, electrolyte panel, and hepatic and renal function profiles. Ultrasound-guided FNAC was performed on both thyroid lobes. Smears showed abundant follicular epithelial cells admixed with lymphocytes and inflammatory cells. The cytological findings were consistent with a benign lesion, favoring HT, and were classified as Bethesda category II (**Figure 3A**).

### 2.3 Surgical pathology and immunohistochemistry

On May 27, 2025, after relevant contraindications were ruled out, the patient underwent bilateral thyroidectomy under general anesthesia. Intraoperative exploration revealed diffuse bilateral thyroid enlargement, with the right lobe measuring approximately 12 × 7 × 5 cm and the left lobe approximately 10 × 6 × 4 cm. Both lobes were firm in consistency. The resected specimen was submitted for intraoperative frozen section analysis. The frozen section report (**Figure 3B**) suggested a thyroid neoplasm, with medullary thyroid carcinoma as the primary consideration, and excluded lymphohematopoietic malignancy. A definitive diagnosis was deferred pending examination of permanent sections with extensive sampling and immunohistochemistry. An additional anterior cervical lymph node was excised, and frozen section analysis suggested metastatic carcinoma (**Figure 3C**). Postoperative routine pathology (**Figure 3D**) indicated a tumor of lymphohematopoietic origin. The final diagnosis of EMP was established based on morphology and immunohistochemical findings. Microscopically, the tissue demonstrated a diffuse infiltrate of plasma cells arranged in solid sheets. The tumor cells exhibited eccentrically located nuclei with marked pleomorphism, irregular contours, and lobulation. A distinct perinuclear halo was also observed. The stroma consisted of a fibrovascular network.

**Figure 4:**
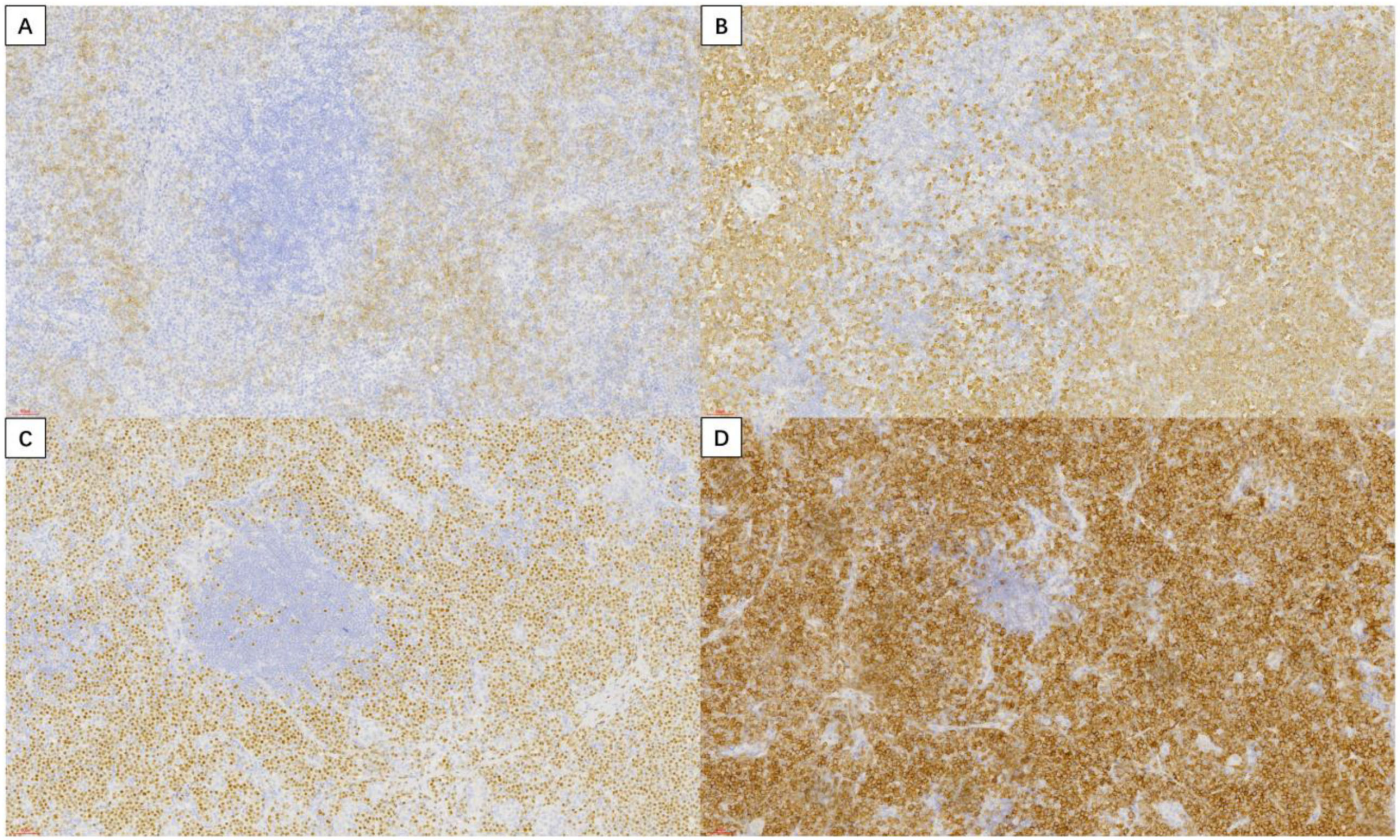
Immunohistochemistry (magnification, ×200): **(A)** CD138 (+, partial); **(B)** Lambda (+); **(C)** MUM-1 (+); **(D)** CD38(+).

Immunohistochemistry (**Figures 4A–D**) showed the following profile: CD20 (-), CD138 (+, partial), Kappa (-), Lambda (+), MUM-1 (+), CD38 (+), CK (-), CD3 (-), CD5 (-), CD21 (+ in FDC meshwork), CD79a (+), CD30 (-), CD10 (-), C-Myc (-), Bcl-2 (+), Bcl-6 (-), Ki-67 (30% +, hot spots), P53 (+, wild-type pattern), PAX-5 (-).

### 2.4 Postoperative management

The patient’s postoperative recovery was uneventful, with resolution of dysphagia and relief of airway compression. No complications were observed. Subsequent bone marrow aspiration and biopsy showed no evidence of bone marrow involvement, confirming the diagnosis of solitary thyroid EMP. The patients were treated with 200 μg of inhibitory thyroxine daily. The hematologist recommended radiotherapy (RT) due to lymph node metastasis, but the patient declined. No additional treatment was administered. At the 6-month follow-up, the patient remained in good condition without recurrence or metastasis.

## 3 Literature review

### 3.1 Methodology

**Figure 5:**
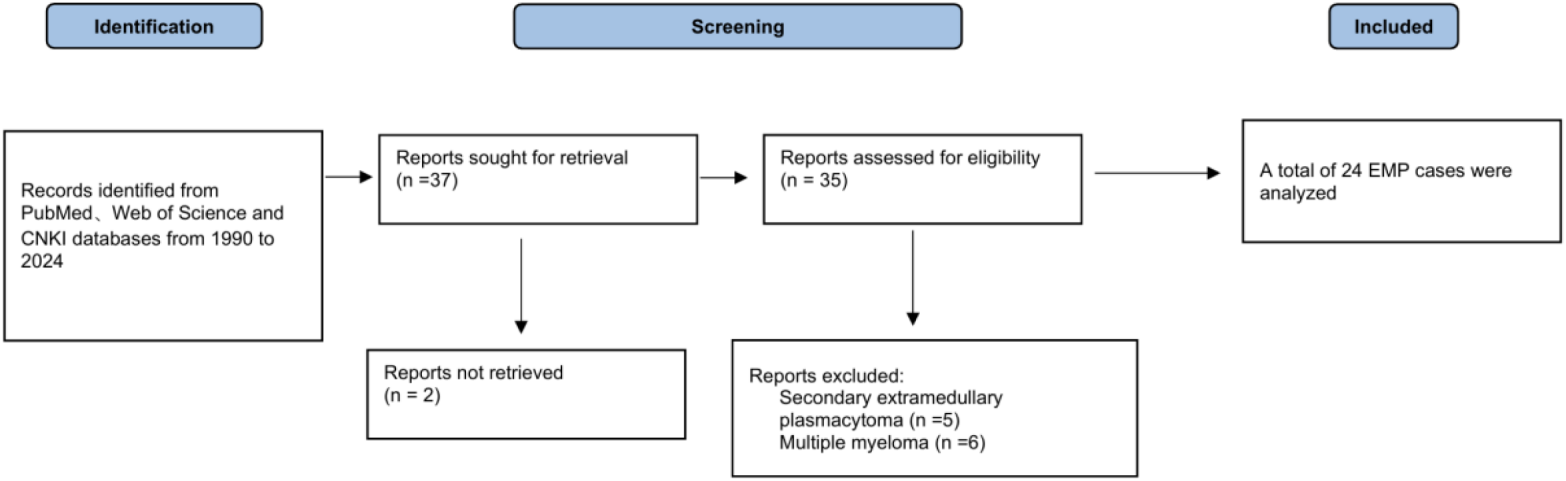
Flow diagram of the literature search and study selection process in the systematic review.

We conducted a systematic literature search of the PubMed, Web of Science, and CNKI databases for studies published between 1990 and 2024. The search terms included “thyroid extramedullary plasmacytoma, “ “primary thyroid plasmacytoma, “ and “thyroid plasmacytoma, “ yielding 37 articles. After screening, 24 cases were included in the analysis. The methodology and selection process for the systematic review are presented in **Figure 5**.

### 3.2 Results

A total of 24 cases of thyroid EMP were reviewed in this study. **Table 1** summarizes the pertinent clinical and pathological profiles, therapeutic interventions, and disease outcomes.

**Table 1:** Summary of clinical characteristics of thyroid EMP cases from 1990 to 2024.

Author	Sex	Age	Clinical presentation	Tracheal compression/deviation	Associated thyroid disease	FNAC Results	Radiotherapy (RT)	Outcome
Zou Xiuli et al. (36)	Female	84	Neck mass	NS	HT	NS	NS	NS
Gui Zhen et al. (37)	Female	73	Neck mass, tracheal deviation	Rightward tracheal deviation	HT	NS	NS	NS
Yin Gang et al. (38)	Male	46	Neck mass	NS	Possibly HT	Suspected HT	Two doses of RT	Disease-free at 3 months
Yao Li et al. (39)	Female	81	Cough, sputum production, shortness of breath	NS	NS	NS	NS	NS
Yan Qinying et al. (40)	Female	74	Neck mass	NS	HT	NS	DT50 Gy/25 fractions	Disease-free at 8 months
Yao et al. (41)	Male	45	Neck mass , dysphagia	tracheal deviation	HT	Thyroiditis	NS	NS
Mertens et al. (42)	Female	78	Neck mass, dyspnea, dysphagia	NS	HT	Suspicious for lymphoma	NS	Disease-free at 17 months
Bhat et al. (28)	Male	60	Neck mass, hoarseness, dysphagia	NS	HT	Strong possibility of medullary carcinoma	NS	NS
Aoyama et al. (33)	Male	68	Neck mass, tracheal deviation	Leftward tracheal deviation	Suspected HT	Unclear benign/malignant distinction	NS	Disease-free at 30 months
Ridal et al. (43)	Female	52	Neck mass	NS	No	Lymphoplasmacytic lymphoma or plasmacytoma	NS	Disease-free at 5 months
Wysocki et al. (44)	Female	46	Anxiety, dizziness, weight loss, decreased appetite	No	(Subclinical hyperthyroidism with toxic multinodular goiter)Hyperthyroidism with toxic multinodular goiter	NS	NS	Disease-free at 9 months
Chesyln-Curtis et al. (45)	Female	78	Neck mass, dysphagia, tracheal deviation	Yes	No	NS	NS	Disease-free at 18 months
Gilani et al. (46)	Female	69	Neck mass	NS	No	Thyroid abscess	NS	Postoperative hypothyroidism (4 wk)
Hasegawa et al. (47)	Female	61	Neck mass, tracheal deviation	Yes	HT, hypothyroidism	NS	Yes	NS
Rubin et al. (48)	Female	76	Neck mass, dyspnea, tracheal deviation	Yes	HT, hypothyroidism	NS	Yes	Disease-free at 20 months
Patten et al. (49)	Female	54	Neck mass	NS	HT	Lymphoma	Yes	Disease-free at 5 months
Hassan et al. (50)	Male	53	Neck mass, tracheal deviation	Yes	No	Thyroiditis; possible plasmacytoma	NS	Disease-free at 8 months
Kuo et al. (51)	Female	19	Neck mass	NS	No	Thyroid lymphoma	No	Disease-free at 36 months
Ohshima et al. (52)	Male	52	Hoarseness	NS	HT	Papanicolaou class II	Yes	Disease-free at 36 months
Kovacs et al. (53)	Female	58	Neck mass, dyspnea	NS	HT	Plasmacytoma	Yes	Disease-free at 72 months(6 years)
Lee et al. (15)	Female	56	Neck mass	NS	HT	Plasmacytoma	No	NS
Fraser et al. (54)	Female	64	Multinodular goiter	NS	Multinodular goiter, HT	Lymphoproliferative process	No	NS
Puliga et al. (55)	Female	74	Painful neck mass, hoarseness	NS	Subclinical hypothyroidism	Suspicious malignant neoplasia	No	Disease-free at 16 months
Refai et al. (56)	Female	71	Neck mass	NS	HT	NS	NS	NS

HT, Hashimoto’s thyroiditis; NS, Not stated.

Thyroid EMP occurs more frequently in elderly women, typically between 55 and 70 years of age. A review of the 24 reported cases demonstrated a clear female predominance, with 18 of 24 patients (75%) being female. Most patients were elderly, with mean and median ages of 62 and 62.5 years, respectively (range: 19–84 years). The disease is rare in younger individuals, with only a single case reported in a 19-year-old patient.

The common clinical features of thyroid EMP include a history of HT and a short duration of symptoms, usually presenting as a rapidly enlarging anterior neck mass, hoarseness, or dysphagia. Most patients presented with an anterior neck mass or diffuse thyroid enlargement, observed in 87.5% of cases. Symptoms related to local compression, such as hoarseness, dysphagia, and dyspnea, were the next most frequent, occurring in approximately half of the cases. The symptoms presented by the two patients included cough accompanied by expectoration, and dizziness accompanied by loss of appetite, respectively.

Of the 23 cases in which thyroid function was tested, five showed normal thyroid function, while the remaining 18 (78.3%) exhibited abnormalities. The most common disorder was HT (16/23, 69.6%), followed by one case of hypothyroidism and one case of hyperthyroidism associated with toxic multinodular goiter.

Establishing a definitive preoperative diagnosis of thyroid EMP is challenging. Histopathological findings may be misinterpreted as medullary thyroid carcinoma or lymphoma. Among the 15 patients in **Table 1** who underwent FNAC, about 33% of the biopsy results indicated the characteristics of lymphoma, 20% of the cases were diagnosed as HT, and 20% of the cases had difficulties in distinguishing benign and malignant lesions. Only 2 cases were diagnosed as plasmacytoma by FNAC. Another case, similar to ours, was misdiagnosed as medullary thyroid carcinoma.

Detailed descriptions of postoperative management are notably scarce in the reported cases of thyroid EMP. Among the cases we reviewed, follow-up data were available for 15 patients, with durations ranging from 1 month to 6 years. The mean follow-up period of EMP was 18.9 months. Outcomes were generally favorable, with the majority of cases experiencing no complications. One author reported an instance of hypothyroidism occurring 4 weeks after surgery, which was potentially attributable to inadequate thyroid hormone supplementation. Up to now, no cases of recurrent thyroid EMP have been documented in the available literature.

## 4 Discussion

### 4.1 Demographics and clinical presentation

From a demographic perspective, EMP typically occurs in middle-aged and older adults, with a median age at diagnosis generally ranging from 55 to 65 years (10). The disease exhibits a distinct gender predisposition, predominantly affecting males, who account for approximately 75% to 86% of cases, resulting in a male-to-female ratio of about 2:1 (11). In contrast to this general pattern, thyroid EMP specifically demonstrates a notable female predominance. For instance, in **Table 1**, women constituted 75% of the reported thyroid EMP patients.

Clinically, patients with thyroid EMP frequently present with a history of HT. Common symptoms include a noticeable neck enlargement, hoarseness, and dysphagia, which often develop over a short period. The increased use of imaging and FNAC has led to more frequent incidental diagnoses of thyroid EMP. Lymph node metastasis is rare, with only three reported cases.

### 4.2 Diagnosis and differential diagnosis

Establishing a definitive preoperative diagnosis of thyroid EMP is challenging. Postoperative immunohistochemistry remains the gold standard for definitive diagnosis. Conventional preoperative examinations, including thyroid ultrasound, contrast-enhanced CT, and FNAC, rarely provide conclusive results. FNAC, in particular, has limited diagnostic value. In this case, the patient underwent FNAC using a puncture needle to collect the main follicular epithelial cells surrounding the lesion. The results showed that the aspirate contained monocytes, predominantly lymphocytes and inflammatory cells, which reflected the characteristic changes of HT. Tumor plasma cells were not present in the aspirate.

There are two reasons underlying the limitations of FNAC in the diagnosis of thyroid EMP. Firstly, the diagnostic value of thyroid FNAC is constrained by its inability to reliably differentiate follicular lesions (12). Consequently, sampling errors are more likely to occur in non−follicular variant thyroid tumors, with an incidence of approximately 61% (13). Secondly, for thyroid EMP, the liquid−based thin−layer smear method demonstrates superior accuracy compared with the manual smear method (14) (15). Lee et al. (16) successfully diagnosed a case of thyroid EMP using liquid−based cytology. Given that plasmacytoma is a hematological malignancy, they concluded that smear methods tend to generate additional isolated cells with non−cohesive characteristics, whereas liquid−based preparation better preserves cell clusters (17).

FNAC is the recommended initial diagnostic method for thyroid nodular disease and is routinely used in clinical practice (18). It is a safe, minimally invasive outpatient procedure with approximately 95% accuracy in differentiating benign from malignant thyroid nodules. However, inadequate sampling or indeterminate cytology can result in false-negative rates of 1–11% (19). In contrast, core needle biopsy (CNB) provides higher diagnostic accuracy by obtaining larger tissue samples. These samples preserve histological architecture, allowing a more precise diagnosis. However, using a larger-gauge needle increases the risk of significant and potentially permanent injury to adjacent vital structures (20). A study by Hahn et al. reported that CNB has significantly higher diagnostic yield than FNAC when thyroid nodules exceed 2 cm in diameter or are classified as TI-RADS grade 4 by ultrasonography (21). Therefore, for rapidly growing thyroid nodules larger than 2 cm with inconclusive FNAC results, CNB may be considered to establish a definitive diagnosis and exclude conditions such as thyroid EMP.

The diagnostic performance of intraoperative frozen section for thyroid lesions is characterized by consistently high specificity (93%–100%) but relatively variable sensitivity (58%–94%) (22). Consequently, in patients with indeterminate FNAC, frozen section is frequently employed for its excellent specificity in confirming malignancy, thereby providing intraoperative justification for the extent of surgical intervention (23). When informative, frozen section findings may help prevent unnecessarily aggressive surgery while simultaneously reducing the risk of subsequent reoperation. However, frozen section examination has certain limitations. Its diagnostic utility is particularly restricted in micropapillary carcinomas and well-differentiated adenocarcinomas, especially those exhibiting a predominantly follicular growth pattern (24). Accordingly, several studies have suggested that careful intraoperative gross evaluation by an experienced surgeon may offer comparable diagnostic value, with frozen section leading to a modification of the surgical plan in only about 5% of cases (25). In this case, the patient’s preoperative FNAC indicated HT. However, the concomitant presence of cervical lymphadenopathy and significant tracheal compression raised strong suspicion of malignancy. To clarify the nature of the lesion and guide the extent of surgery, intraoperative frozen section examination was performed. Unfortunately, diagnosing EMP of the thyroid on frozen section is challenging, as this technique is primarily used to differentiate thyroid follicular adenoma from carcinoma. Therefore, frozen section is not recommended for confirming thyroid EMP.

The primary reason for misdiagnosis in this disease is related to morphological pitfalls. Microscopically, EMP cells typically exhibit a plasmacytoid morphology, characterized by eccentric nuclei, eosinophilic cytoplasm, intranuclear inclusions, and nuclear grooves. Clusters of Hurthle cells and amyloid deposits were also identified, closely resembling the cellular morphology and stromal amyloid deposits of medullary thyroid carcinoma (14). Additionally, the diffuse infiltrative pattern of lymphocytes is easily misdiagnosed as lymphoma (26).

However, immunohistochemical features clearly differentiate EMP from these entities. **Table 2** summarizes the identification points of the three diseases. EMP typically expresses CD138, CD38, kappa light chain, lambda light chain, MUM-1, and CD79a, while being negative for CD45, CD19, and CD20. In contrast, lymphoma cells are positive for CD45, CD19, and CD20 (27). Medullary thyroid carcinoma usually expresses calcitonin and CEA but is negative for thyroglobulin (Tg) and CD45 (28). Therefore, when FNAC specimens contain atypical plasmacytoid cells or unclassified cellular populations, immunohistochemistry with plasma cell markers should be considered to minimize the risk of misdiagnosis.

**Table 2:** Compared with medullary carcinoma and lymphoma, the prominent features of thyroid EMP are as follows.

Identification points	Thyroid EMP	Medullary thyroid carcinoma	Thyroid lymphoma
Tissue origin	Plasma cells	Parafollicular cells of the thyroid	Lymphocytes
Pathology	Diffuse infiltration of tumor cells with eccentric nuclei, perinuclear hof, and basophilic cytoplasm; arranged in solid sheets.	Tumor cells arranged in nests/sheets, separated by fibrous stroma often containing amyloid deposits.	Lymphocytes diffusely infiltrated, and cell morphology varied with lymphoma subtypes.
Key immunohistochemical markers	Positive:​ CD138, CD38, MUM-1, CD79a, MUM-1; Restricted expression of Lambda or Kappa light chain; Negative:​ CD45, CD19, CD20, PAX-5, TTF-1, TG.	Positive:​ Calcitonin, CEA, Syn, CgA; Negative:​ TG, CD 45.	Positive: CD45, CD20, CD 19, Pax-5; Negative: CD138, CK, TG
Serum markers	No	Serum calcitonin and CEA are elevated	No

### 4.3 Association between thyroid EMP and HT

The combined rate of thyroid EMP and HT is much higher than the probability of accidental occurrence. Although no definitive studies have clarified the pathogenic mechanism linking thyroid EMP and HT, many researchers suggest that chronic antigenic stimulation within the inflammatory microenvironment of HT may contribute. Thyroid lymphocytes are present under various pathological conditions, and their presence is especially prominent in autoimmune diseases (29). In HT, inflammation with dense lymphocyte infiltration can alter the antigens expressed on thyroid follicles, thus triggering an abnormal immune response (30). In this setting, immune cells and cytokines such as IL-1β, IL-6, and TGF-β can induce aberrant B-cell activation and proliferation, leading to clonal plasma cell expansion and subsequent EMP formation (30). This indicates that patients with long-standing HT represent a high-risk group for thyroid EMP. Clinicians should therefore recognize that rapid thyroid enlargement in patients with HT may not solely indicate disease progression but could suggest a neoplastic lesion such as EMP.

### 4.4 Treatment and follow-up

Thyroid EMP typically remains localized within the thyroid gland, with metastasis being uncommon. Due to its rarity and insidious progression, no consensus has yet been established regarding the optimal treatment approach. In previous studies, most cases were treated surgically, although some patients received RT alone or in combination with surgery (31). As shown in **Table 1**, 30.4% of patients received local RT. This case exhibited no symptoms suggestive of thyroid EMP before surgery. If the patient had been diagnosed with EMP preoperatively, RT alone could have been sufficient. RT is the most common treatment for EMP because of its high radiosensitivity (32). The European Hematology Expert Panel recommends local fractionated radiotherapy as the primary treatment for most EMP cases. Typically, 40–50 Gy is delivered over about four weeks in daily fractions of 1.8–2.0 Gy. The treatment field should include all involved tissues with at least a 2 cm margin of surrounding normal tissue (3). This regimen aligns with histologically supported dose recommendations, as radiotherapy doses above 40 Gy achieve local control rates as high as 94% (33). Research by Sasaki et al. (34)indicates that combined modality therapy, involving surgery and radiotherapy, significantly improves overall survival. This combined approach may represent the optimal strategy for managing head and neck EMP.

To date, no cases of recurrence or metastasis have been reported, and the prognosis for thyroid EMP is generally favorable. Janjetovic et al. (35)reported a 12-year survival rate of approximately 75% for primary EMP. In our literature review, follow-up data were available for 15 patients, but the duration ranged widely from 1 month to 6 years (mean: 18.9 months). Most patients experienced no complications. Only one patient reportedly developed hypothyroidism 4 weeks postoperatively, likely due to inadequate thyroid hormone supplementation. Our patient continues long-term follow-up to monitor for potential recurrence and assess outcomes.

## 5 Conclusion

This report presents a case of thyroid EMP initially misdiagnosed as uncomplicated HT, and, for the first time, comprehensively documents the complete diagnostic and therapeutic process. Moreover, to our knowledge, this study provides the most extensive systematic review of the literature to date, incorporating all previously published thyroid EMP cases. We conducted a detailed discussion of clinical manifestations, pathological features, and pathogenesis, and summarized both prognosis and key aspects for pathological differentiation. The pathogenesis of thyroid EMP is likely related to chronic antigenic stimulation within the inflammatory microenvironment characteristic of HT. Therefore, when HT patients exhibit rapid thyroid enlargement, the liquid−based thin−layer smear method should be used during fine−needle aspiration to increase diagnostic accuracy. When FNAC reveals atypical plasma cells or unclassifiable cells, clinicians and pathologists need to be alert to neoplastic lesions such as EMP. Ancillary immunohistochemical testing for plasma cell markers should be performed routinely to minimize the risk of misdiagnosis. Future research should further clarify the pathogenesis of thyroid EMP, develop expert consensus regarding surgical and postoperative management strategies, and collect more extensive long-term follow-up data.
